# Efficacy and safety of thermal ablation for Bethesda III/IV thyroid nodules: a retrospective study

**DOI:** 10.3389/fendo.2025.1572535

**Published:** 2025-05-02

**Authors:** Song Li, Ying Wei, Zhen-long Zhao, Li-li Peng, Yan Li, Ming-an Yu

**Affiliations:** Department of Interventional Ultrasound Medicine, China-Japan Friendship Hospital, Beijing, China

**Keywords:** thyroid nodule, thermal ablation, Bethesda classification, ultrasonography, minimally invasive

## Abstract

**Objectives:**

To evaluate the efficacy and safety of thermal ablation (TA) for the treatment of Bethesda III and IV thyroid nodules.

**Materials and methods:**

A retrospective analysis was conducted on 154 patients with Bethesda III (n = 82) and IV (n = 72) thyroid nodules treated with microwave or radiofrequency ablation between December 2016 and October 2023. Patients were followed for a median of 19 months. Outcomes assessed included nodule volume reduction rate (VRR), complications, and disease progression.

**Results:**

The median VRR at 12 months was 97% (Bethesda III) and 88% (Bethesda IV), increasing to 100% and 96% by 36 months, respectively, with no significant differences between groups. No major complications were encountered, minor complications occurred in 4 patients (4/154, 2.6%), including 3 cases of transient hoarseness and 1 case of neck pain, resolving spontaneously. Disease progression (3/154, 1.9%) occurred in both groups, with new tumor in each group, no other disease progression occurred.

**Conclusion:**

Thermal ablation could be a safe, effective, and minimally invasive alternative to surgery for Bethesda III/IV thyroid nodules, achieving substantial volume reduction with minimal complications.

## Introduction

Thyroid nodules, commonly detected on ultrasound, are classified by the Bethesda Thyroid Cytopathology Reporting System. Bethesda III/IV nodules, representing atypia/follicular lesions of undetermined significance (AUS/FLUS) and follicular neoplasms, account for 10–30% of nodules with a malignancy risk of 5–30%. Managing these nodules is challenging due to diagnostic uncertainty (1–3).

Current guidelines recommend ultrasound evaluation and follow-up for Bethesda III/IV nodules, supplemented by repeated fine-needle aspiration (FNA) cytology or molecular testing when available, to assess malignancy risk. Diagnostic surgery is often the definitive approach (4–6). However, regular follow-up and repeated FNA may impose significant psychological and financial burdens on patients. Moreover, surgery carries risks, including hypothyroidism (9.9%–14.5%), parathyroid damage (temporary: 8.3%–54.4%; permanent: 1.6%–5.9%), and recurrent laryngeal nerve injury (temporary: 2.0%–2.8%; permanent: 0.5%–1.4%) (7–10). Additionally, a recent study reported that up to 68% of surgeries for Bethesda III/IV nodules without molecular testing were unnecessary, in other words, an overtreatment (11). These challenges highlight the need for a minimally invasive, effective, and low-risk alternative to surgery for managing indeterminate thyroid nodules.

Thermal ablation (TA) techniques, such as microwave (MWA) and radiofrequency ablation (RFA), have emerged as promising alternatives to surgery for benign thyroid nodules (BTNs) and selective papillary thyroid carcinomas (PTC) due to their minimally invasive nature, preservation of thyroid function, and low complication rates (12–15). Some guidelines recommend thermal ablation as an alternative treatment for BTNs or PTC (16, 17). Since TA could be used to safely and effectively manage benign and malignant thyroid tumors, could it be used to manage indeterminate thyroid nodules?

This study aims to evaluate the safety and efficacy of thermal ablation as a minimally invasive treatment option for indeterminate (Bethesda III/IV) thyroid nodules, addressing current gaps in clinical research.

## Materials and methods

This retrospective study was approved by the Institutional Review Board of the China-Japan Friendship Hospital (Ethics Approval No.: S2019-283-02). Written informed consent for the ablation procedure was obtained from all patients. The ethics committee waived the requirement for written consent for data publication, as all patient data were anonymized, and confidentiality was strictly maintained.

### Patient

**Figure 1:**
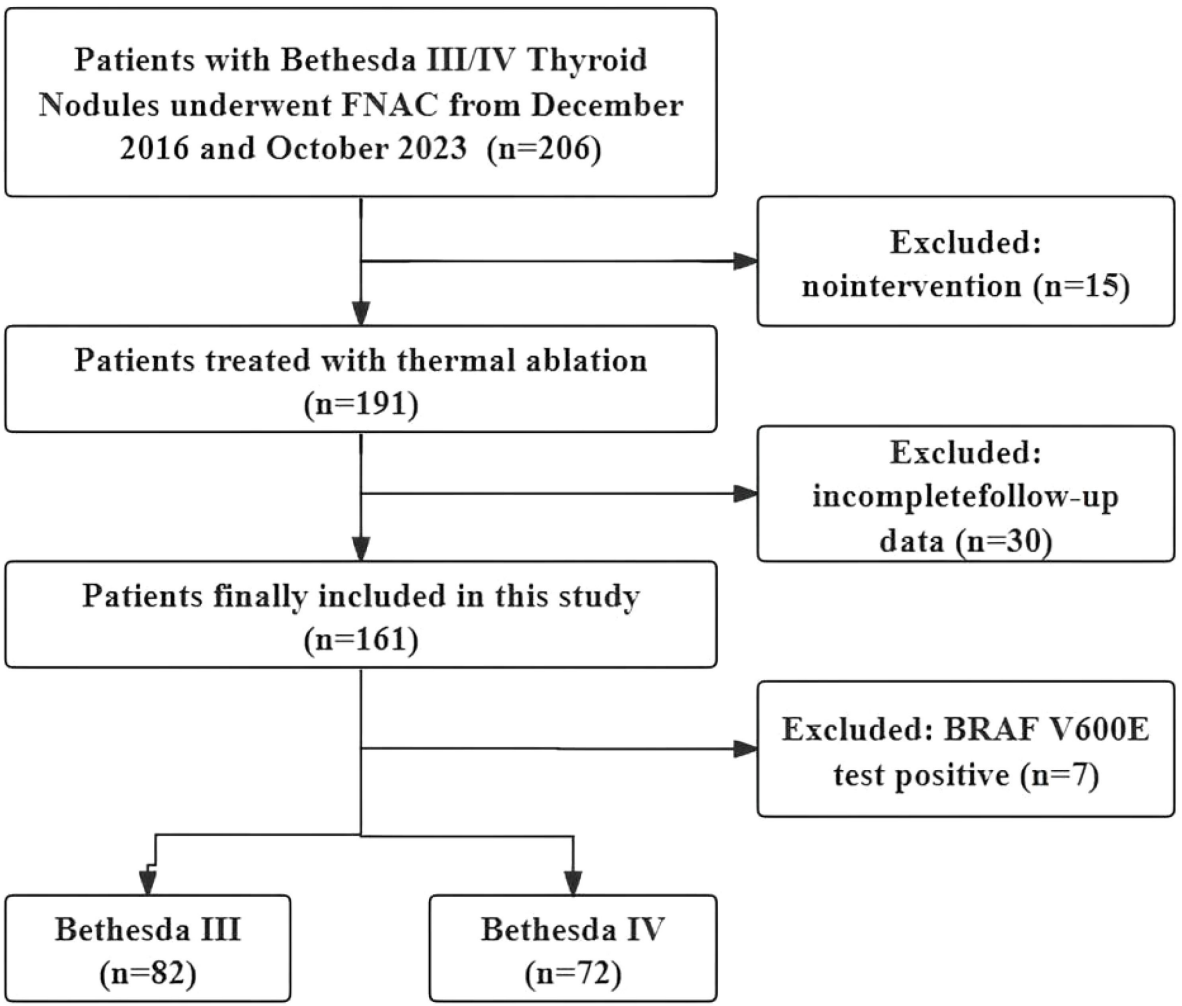
Study flowchart.

Clinical data from patients with thyroid nodules treated with TA between December 2016 and October 2023 were retrospectively analyzed. The inclusion criteria were as follows: (i) a diagnosis of Bethesda III or IV confirmed by US-guided fine-needle aspiration biopsy, (ii) refusal of or ineligibility for surgery and (iii) a follow-up time of at least 12 months. The exclusion criteria were as follows: (i) coexisting other thyroid tumors, such as PTC or hyperfunctioning thyroid adenoma; (ii) other malignant diseases; (iii) follow-up less than 12 months; (iv) incomplete clinical data; (v) BRAF V600E positive and (vi) prior hemithyroidectomy. The study flow chart of the enrolled cases is shown in **Figure 1**.

### Pre-ablation assessment

Prior to ablation, all patients underwent neck US, neck and chest computed tomography (CT), US-guided FNA, BRAF V600E mutation testing, and laboratory tests including thyroid function, routine blood test and coagulation function. Routine US examinations meticulously documented nodule dimensions along three meridians, location, sonographic traits, and tumor vascularity. The volume of the nodule was determined using the equation *V* = π*abc*/6, where ‘V’ is volume, ‘a’ denotes the maximum diameter on US (MD), and ‘b’ and ‘c’ represent the other two perpendicular diameters. In cases of multiple thyroid nodules, only data from the largest nodule was considered for analysis (18). The medical records of patients, including the results from US examinations, CT images, and laboratory tests, were independently reviewed by three physicians with more than three years of experience in thyroid ablation (18, 19).

### TA procedure

All ablation procedures were conducted by three radiologists with over 3 years of experience. Utilizing either the MWA (Intelligent Basic Type Microwave Tumor Ablation System, Nanjing ECO Microwave System) and RFA (Cooltip Radiofrequency Ablation System [Covidien]) were performed under local anesthetic. These procedures were performed under US guidance, with patients in a supine position and their necks fully exposed. Continuous monitoring was ensured through electrocardiogram and pulse oximetry (13, 20).

Prior to TA, local anesthesia was applied at the puncture site with topical 1% lidocaine. For protecting vital structures such as the trachea, recurrent laryngeal nerve, and major cervical vessels near the thyroid, the hydrodissection technique was employed basing on the fascial spaces to achieve an isolation distance of at least 4 mm. Under US direction, the ablation antenna was precisely inserted into the thyroid nodules. We employed conformal ablation using the moving-shot technique to sequentially inactivate the nodules with power settings of 30 W for MWA and 40 to 60 W for RFA. The termination for ablation was determined when the transient hyperechoic echotexture enveloped the entire nodule. Subsequently, CEUS examination verified the completeness of the ablation and ensured the therapeutic effect (18).

**Figure 2:**
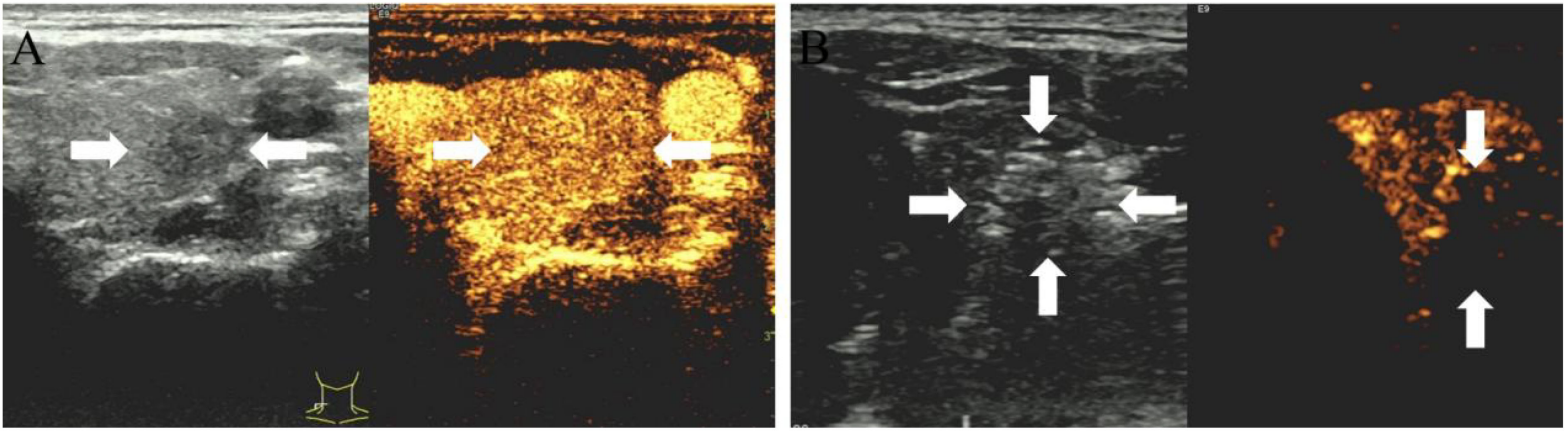
**(A)** 35-year-old male with a Bethesda IV thyroid nodule underwent thermal ablation. Pre-procedural conventional ultrasonography revealed a hypoechoic nodule (white arrows) in the left thyroid lobe (white arrows). Contrast-enhanced ultrasound prior to ablation demonstrated a hypoenhanced pattern during the arterial phase (white arrows). **(B)** Postablation contrast-enhanced ultrasound image shows no enhancement in the original tumor zone (white arrow).

**Figure 3:**
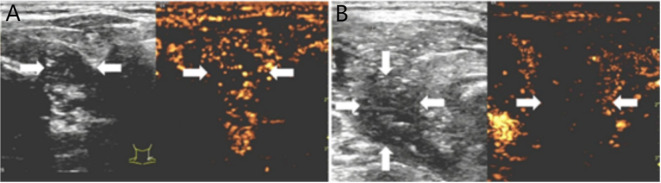
**(A)** 50-year-old female with a Bethesda III thyroid nodule underwent thermal ablation. Pre-procedural conventional ultrasonography revealed a hypoechoic nodule (white arrows) in the right thyroid lobe (white arrows). Contrast-enhanced ultrasound prior to ablation demonstrated a hypoenhanced pattern during the arterial phase (white arrows). **(B)** Postablation contrast-enhanced ultrasound image shows no enhancement in the original tumor zone (white arrow).

A successful technique is defined by the complete ablation of the neoplasm, where the non-enhanced ablation zone fully encompasses the target neoplasm. Otherwise, further ablation should be carried out immediately. Post-procedurally, vocal cord function was reassessed by US, fiberoptic laryngoscopy assessments were conducted as required to ensure a comprehensive evaluation. Patients were closely monitored for any potential complications (12). Representative US images of the Bethesda III and IV thyroid nodules ablation procedure are depicted in **Figures 2**, **3**.

### Post-ablation assessment and follow-up

Post-ablation monitoring was scheduled at 1, 3, 6, 9, and 12 months within the first year, followed by semiannual evaluations. The examinations included US, thyroid function tests, and annual chest CT scans. Post-ablation US played a crucial role in tracking tumor dimensions and volume, thus facilitating the assessment of ablation success, recurrence, and LNM. Chest CT scans were instrumental in identifying possible lung metastases. The volume reduction ratio (VRR) was computed using the formula: VRR(%)= [(initial volume-final volume)/initial volume] × 100% (12, 18).

Adverse events were classified as either major or minor complications, or side effects, and were meticulously documented according to the Society of Interventional Radiology (SIR) Standards of Practice Committee classification. Disease progression was defined as the occurrence of new thyroid nodules, local recurrence of the ablated lesion, or LNM during follow-up (21).

### Statistical analysis

All statistical analyses were performed using SPSS version 26.0. Continuous variables were expressed as mean ± standard deviation (SD) or median (interquartile range, IQR), depending on the data distribution. Categorical variables were presented as frequencies and percentages. Normality of the data was assessed using the Shapiro-Wilk test. Comparisons between groups were performed using the independent-samples t-test or Mann-Whitney U test for continuous variables and the chi-square test or Fisher’s exact test for categorical variables. A p-value of <0.05 was considered statistically significant.

## Results

There were a total of 154 cases enrolled in the present study, including 82 cases in the Bethesda III group and 72 cases in the Bethesda IV group. Baseline characteristics were similar between the two groups of patients, as summarized in **Table 1**. The median age (Bethesda III vs. Bethesda IV = 50 vs. 47 years, *p* = 0.220), sex distribution (*p* = 0.889), ablation time (*p* = 0.345), power (*p* = 0.232), and follow-up times (*p* = 0.087) showed no significant differences.

**Table 1:** Baseline characteristics of cases.

Characteristic	Bethesda III group (N = 82)	Bethesda IV group (N = 72)	*p*-value
Age (years)	50 (37, 57)	47 (33, 57)	0.220
Sex			0.889
Female	63 (76.8%)	56 (77.8%)	
Male	19 (23.2%)	16 (22.2%)	
Technique of TA*			0.200
Microwave ablation	56 (68.3%)	42 (58.3%)	
Radiofrequency ablation	26 (31.7%)	30 (41.7%)	
Margin			0.574
Irregular	19 (23.2%)	14 (19.4%)	
Smooth	63 (76.8%)	58 (80.6%)	
Calcification			0.065
None	70 (85.4%)	68 (94.4%)	
Yes	12 (14.6%)	4 (5.6%)	
Location			0.339
Isthmus	1 (1.2%)	0 (0%)	
Left lobe	40 (48.8%)	29 (40.3%)	
Right lobe	41 (50.0%)	43 (59.7%)	
CEUS			0.005
Hyper-enhancement	45 (54.9%)	56 (77.8%)	
Hypo-enhancement	30 (36.6%)	10 (13.9%)	
Iso-enhancement	7 (8.5%)	6 (8.3%)	
Ablation time (s)	150 (88, 226)	179 (90, 349)	0.345
Ablation power (W)	30 (30, 60)	30 (30, 60)	0.232
Follow-up (month)	19 (12, 30)	13 (10, 25)	0.087

*TA, thermal ablation.

Successful thermal ablation was achieved in all of the enrolled cases in a one session. The techniques (MWA and RFA) were evenly distributed between the groups (MWA vs. RFA = 63.64% vs. 36.36%, *p* = 0.200). Calcification was slightly more common in the Bethesda III group (14.6% vs. 5.6%, *p* = 0.065), while smooth margins were nearly identical in both groups (*p* = 0.574). Nodules were predominantly located in the left and right lobes of the thyroid. Hyper-enhancement (Bethesda III vs. Bethesda IV = 54.9% vs. 77.80%, *p* = 0.005) was the most common CEUS finding in both groups.

### Changes in nodule size after thermal ablation for Bethesda III/IV thyroid nodules

Pre-ablation volumes were similar between the two groups (Bethesda III vs. Bethesda IV = 4.2 ± 7.1 cm² vs. 5.3 ± 8.4 cm², *p* = 0.371). After ablation, the volumes of ablation zone were comparable between Bethesda III and IV thyroid nodules at all evaluated time points (1, 3, 6, 9, 12, 18, 24, and 36 months, as shown in **Table 2**). Although post-ablation volume reductions were observed, no significant differences were found at 1 month (3.6 ± 4.9 cm² vs. 3.9 ± 6.5 cm², *p* = 0.766) or 3 months (2.09 ± 3.38 cm² vs. 2.06 ± 3.67 cm², *p* = 0.963). This pattern remained consistent during subsequent follow-ups at 6, 9, 12, 18, 24, and 36 months, with numerical differences observed but no statistically significant findings (all *p* > 0.05).

**Table 2:** Changes in nodule size after thermal ablation.

Follow-up time	Volume (cm^3^)			VRR* (%)		
	Bethesda III (n=86)	Bethesda IV (n=75)	p-value	Bethesda III (n=86)	Bethesda IV (n=75)	p-value
Before TA	4.2 ± 7.1	5.3 ± 8.4	0.371	NA	NA	NA
1-month	3.6 ± 4.9	3.9 ± 6.5	0.766	0 (-600, 0)	0 (-100, 0)	0.464
3-months	2.09 ± 3.38	2.06 ± 3.67	0.963	30 (-90, 60)	40 (0, 70)	0.252
6-months	2.00 ± 2.91	1.62 ± 2.62	0.525	50 (15, 86)	56 (32, 80)	0.802
9-months	0.80 ± 1.57	1.52 ± 2.91	0.234	88 (57, 99)	78 (58, 97)	0.793
12-months	0.52 ± 1.06	0.82 ± 1.16	0.220	97 (72, 100)	88 (56, 100)	0.157
18-months	0.49 ± 1.22	0.62 ± 0.90	0.685	96 (81, 1.00)	89 (81, 100)	0.587
24-months	0.50 ± 1.13	0.13 ± 0.31	0.138	100 (90, 100)	100 (99, 100)	0.616
36-months	0.13 ± 0.28	0.60 ± 1.37	0.398	100 (98, 1.00)	96 (61, 100)	0.460

*VRR, volume reduction ratio. *NA, Not Available.

VRR was also comparable between Bethesda III and IV nodules throughout the study period. At 1 month, median VRR was identical between the groups (0% vs. 0%, *p* = 0.464). By 3 months, median VRR increased slightly in both groups (30% vs. 40%, *p* = 0.252), and this trend persisted through 6, 9, 12, 18, 24, and 36 months. At 12 months, median VRR reached 97% for Bethesda III and 88% for Bethesda IV nodules (*p* = 0.157), and at 36 months, it reached 100% and 96%, respectively (*p* = 0.460). These results indicate a consistent and comparable volume reduction in both groups, irrespective of Bethesda classification.

**Figure 4:**
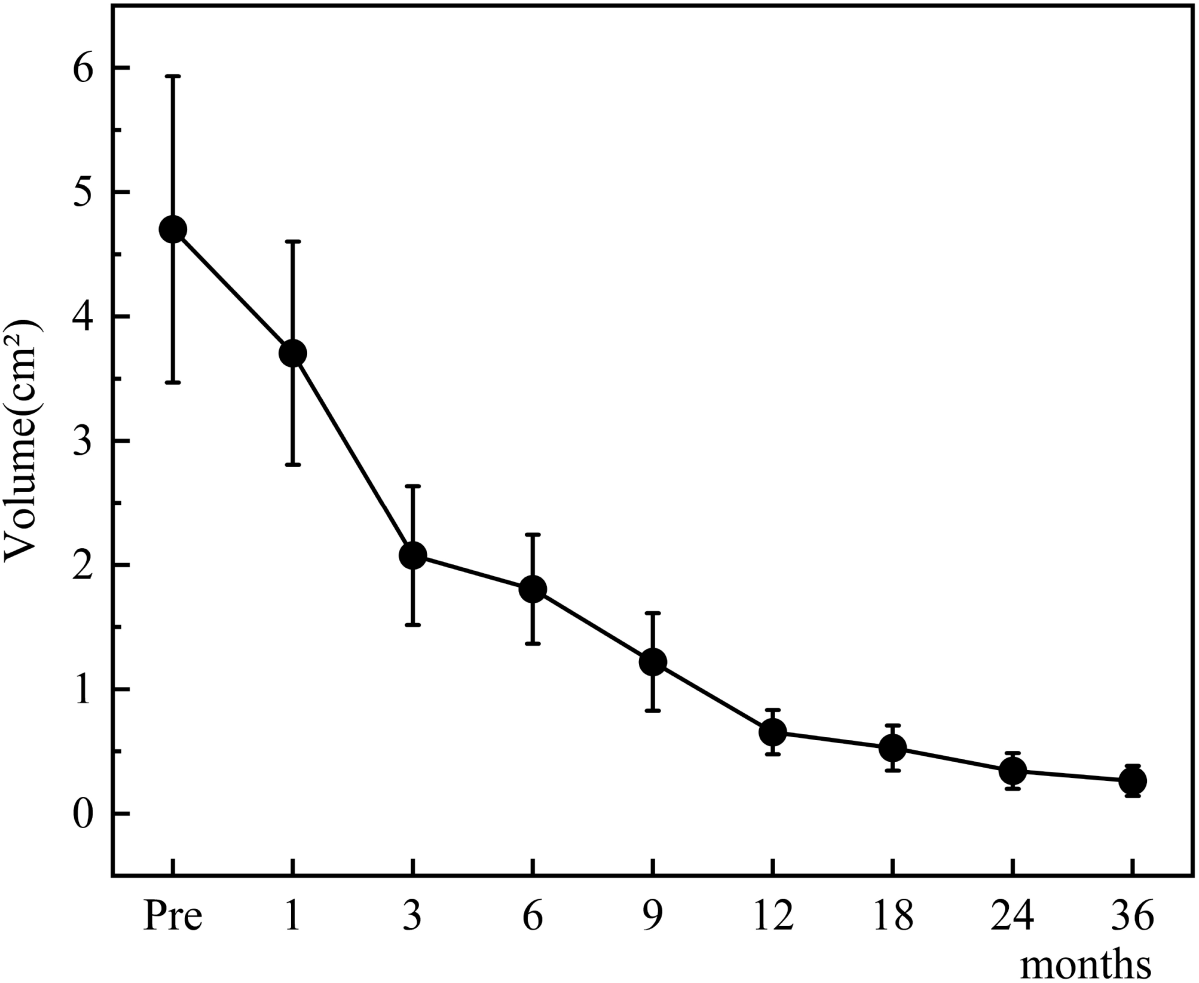
Changes in tumor volume after TA for Bethesda III/IV thyroid nodules at each follow-up point.

**Figure 5:**
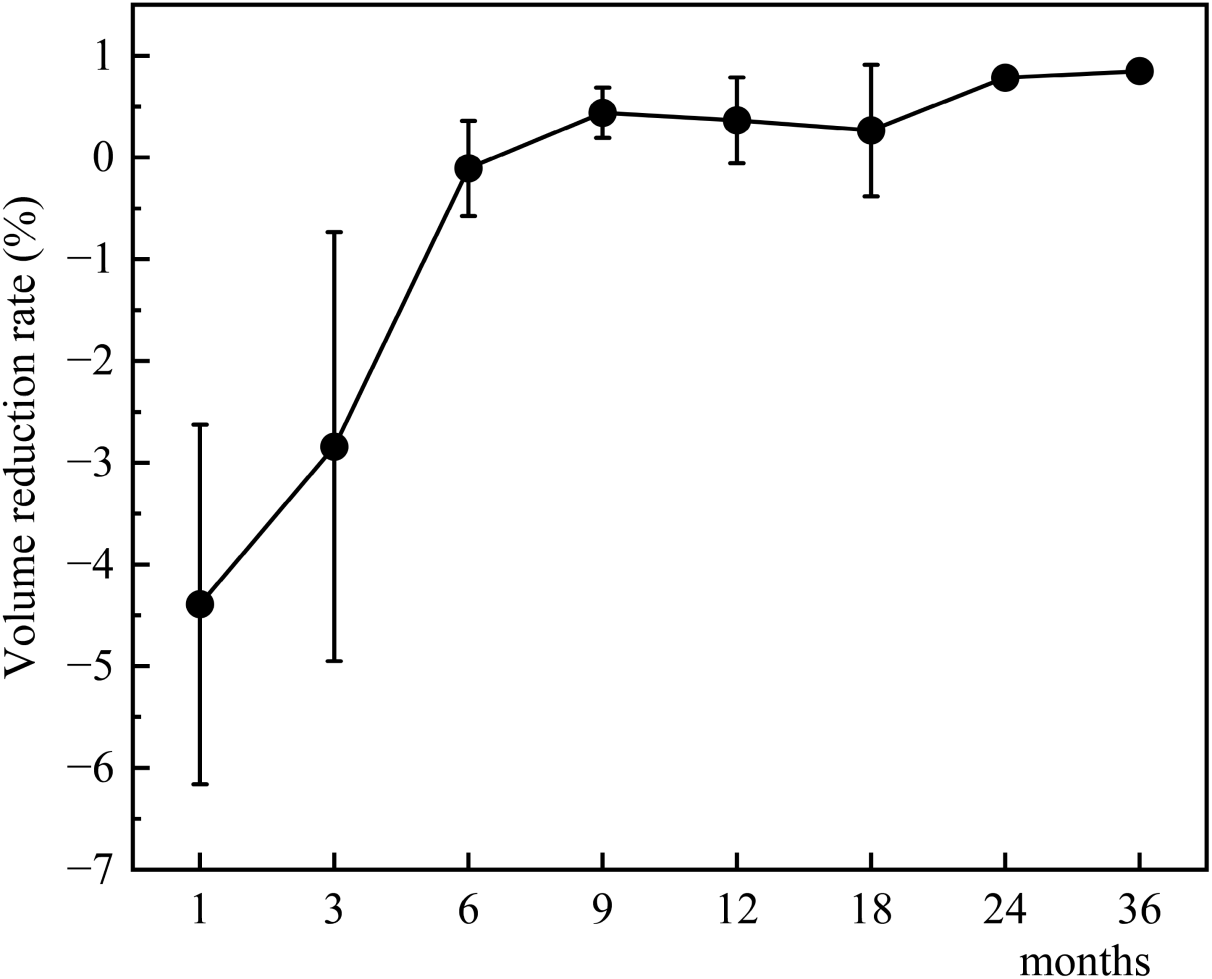
Changes in tumor volume reduction rate after TA for Bethesda III/IV thyroid nodules at each follow-up point.

The changes in tumor volume and VRR at each follow-up point after TA for Bethesda III/IV thyroid nodules are shown in **Figures 4**, **5**.

### Complications

In the Bethesda III group, three patients (3.7%, 3/82) experienced transient hoarseness, which resolved spontaneously within 3 months without treatment. In the Bethesda IV group, one patient (1.4%, 1/72) reported transient neck pain, which resolved the following day without intervention. No other complications were observed in either group. The overall complication rate was low and comparable between the groups (Bethesda III: 3.7% vs. Bethesda IV: 1.4%, *p* = 0.707).

### Disease progression

In the Bethesda III group, one patient (1.2%, 1/82), a 58-year-old woman with a BRAF V600E-negative thyroid nodule, developed a new nodule one year after ablation. This nodule was diagnosed as PTC via FNA. In the Bethesda IV group, two patients (2.8%, 2/72)—a 26-year-old man and a 50-year-old woman, both with BRAF V600E-negative thyroid nodules—developed new nodules one year and two years after ablation, respectively. These nodules were also diagnosed as PTC via FNA. All patients underwent additional ablation therapy, which successfully eradicated the new lesions. No further disease progression, including new tumors, residual disease, or metastatic lymph nodes, was observed during subsequent follow-up. The overall disease progression rate was low and showed no significant difference between the two groups (Bethesda III: 1.2% vs. Bethesda IV: 2.8%, *p* = 909).

## Discussion

Diagnostic surgery is traditionally used to clarify the nature of indeterminate thyroid nodules but carries significant risks, including scarring and hypothyroidism, parts of them may require lifelong thyroxine replacement therapy. Surgery is also associated with longer recovery times and unnecessary interventions, as many operated nodules are ultimately benign. In contrast, TA offers a minimally invasive alternative with advantages such as thyroid preservation, lower complication rates, and shorter recovery times (22–24).

The recurrence rate in the present study is 1.86%. These findings align with previous studies. For instance, Zhou et al. reported a 2.7% recurrence rate in Bethesda IV nodules treated with MWA, with no cases of distant metastases (18). Similarly, RFA studies have shown recurrence rates of 0% to 33% (22, 25–27), depending on the follow-up duration. It is worth noting that the disease progression in the present study consisted exclusively of new tumors. All of these new tumors were confirmed as PTCs through FNA and were successfully treated with additional TA. It is speculated that there is no relationship between the treated Bethesda III/IV thyroid tumors and the new PTC tumors. In other words, no disease progression was directly related to the treated Bethesda III/IV thyroid tumors. The above results demonstrate a promising outcome for TA of Bethesda III/IV thyroid tumors, even though a few of them may be malignant.

BRAF V600E testing was performed to stratify malignancy risk, as BRAF-V600E positive nodules have higher malignancy potential (28). Although current guidelines do not universally exclude BRAF-positive Bethesda III/IV nodules, the cytological features of these nodules remain inherently indeterminate (29). Larger cohort studies and more comprehensive molecular profiling are needed in the future to further refine the risk stratification system for Bethesda III/IV nodules. Based on current evidence and expert consensus, thermal ablation should be considered only for carefully selected patients with Bethesda class III or IV nodules to optimize treatment outcomes and minimize potential risks.

Because most cases of Bethesda III/IV thyroid tumors present with compressive symptoms or appearance-related anxiety. VRR is an important indicator of ablation efficacy. In our study, In our study, VRR reached 97% (Bethesda III) and 88% (Bethesda IV) at 12 months, increasing to 100% and 96% respectively at 36 months. These results are consistent with previous findings reported by Dong et al. and Lin et al (22, 26), who observed similar VRRs at 12 months. Longer follow-up studies, such as those by Ha et al., have demonstrated VRRs exceeding 90% at 24 months, further reinforcing the long-term efficacy of TA (25).

TA demonstrated a strong safety profile, with only four minor complications: transient hoarseness in three Bethesda III patients and transient neck pain in one Bethesda IV patient. All complications resolved spontaneously, resulting in a low complication rate of 2.6%. This rate is significantly lower than surgical complication rates, which range from 2.5% to 8.1% (10, 30). No cases of hypothyroidism were encountered in the present study, highlighting TA’s ability to avoid postoperative hypothyroidism and preserve long-term thyroid function.

Several limitations should be acknowledged in this study. First, its retrospective, single-center design inherently introduces selection bias and restricts the generalizability of the findings. Second, the absence of patient-reported outcomes (PROs), particularly validated instruments such as compressive symptom scales or standardized cosmetic assessments, limits the ability to evaluate treatment effectiveness from the patient’s perspective. Third, the relatively small sample size with 36-month follow-up data (n = 154) and a short median follow-up duration (19 months) may not sufficiently capture rare complications or long-term recurrence patterns—both crucial parameters for assessing the clinical value of thermal ablation.

These methodological constraints underscore the need for future rigorously designed investigations. Priority should be given to multicenter prospective trials with larger cohorts and extended surveillance periods (≥5 years). Such studies should systematically incorporate validated thyroid-specific PRO instruments, notably the ThyPRO (Thyroid-Related Patient-Reported Outcome) questionnaire, while concurrently evaluating potential prognostic factors. A multidimensional approach integrating clinical, imaging, and patient-centered outcomes would facilitate the generation of robust evidence to optimize thermal ablation protocols in thyroid cancer management.

## Conclusion

Thermal ablation is a safe, effective, and minimally invasive alternative to surgery for Bethesda III and IV thyroid nodules. It offers significant volume reduction, minimal complications, and thyroid preservation, making it a promising option for broader clinical application. Long-term follow-up is warranted in the future study to evaluate its role in the comprehensive management of indeterminate thyroid nodules.

## Data Availability

The original contributions presented in the study are included in the article/supplementary material. Further inquiries can be directed to the corresponding author.
